# Hsa_circ_0044226 knockdown attenuates progression of pulmonary fibrosis by inhibiting CDC27

**DOI:** 10.18632/aging.103543

**Published:** 2020-07-24

**Authors:** Fei Qi, Yong Li, Xue Yang, Yanping Wu, Lianjun Lin, Xinmin Liu

**Affiliations:** 1 Department of Geriatrics, Peking University First Hospital, Beijing 100034, China

**Keywords:** Idiopathic pulmonary fibrosis, circular RNAs, CDC27, bioinformatics analysis

## Abstract

Idiopathic pulmonary fibrosis (IPF) is a chronic and progressive lung disorder. Here, we performed a bioinformatics analysis using the GSE102660 dataset from the Gene Expression Omnibus database to identify differentially expressed circRNAs (DEcircRNAs) in tissues from IPF patients and healthy controls. The results identified 45 DEcircRNAs, among which expression of hsa_circ_0044226 was markedly higher in lung tissues from IPF patients than from healthy controls. Knocking down hsa_circ_0044226 expression using a targeted shRNA inhibited TGF-β1-induced fibrosis in RLE-6TN cells and in a bleomycin-induced mouse model of IPA. The diminished TGF-β1-induced fibrosis was associated with upregulated expression of E-cadherin and downregulated expression of α-SMA, collagen III and fibronectin 1, as well as with reduced expression of CDC27, suggesting inhibition of epithelial-to-mesenchymal transition (EMT). All of those effects were reversed by overexpression of CDC27. This suggests CDC27 overexpression abolishes the antifibrotic effect of hsa_circ_0044226 knockdown through activation of EMT. Furthermore, hsa_circ_0044226 knockdown decreased the expression of CDC27 in BLM-induced pulmonary fibrosis mouse model. Collectively then, these findings indicate that downregulation of hsa_circ_0044226 attenuates pulmonary fibrosis *in vitro* and *in vivo* by inhibiting CDC27, which in turn suppresses EMT. This suggests hsa_circ_0044226 may be a useful therapeutic target for the treatment of IPF.

## INTRODUCTION

Pulmonary fibrosis is a chronic, progressive lung disease [1]. Pulmonary fibrotic diseases include sarcoidosis, hypersensitivity pneumonitis, radiation-induced fibrosis, and idiopathic pulmonary fibrosis (IPF) [2]. Of them, IPF, which is characterized by inexorable decline in lung function, stiffening and fibrosis of the lung parenchyma, and progressive respiratory failure, ultimately leading to death, has the poorest prognosis [3, 4]. The 3-5-year survival rate among patients with IPF is only 50% after diagnosis [5]. The mechanisms underlying IPF are not fully understood, though environmental and occupational exposures, cigarette smoking, and viruses are all risk factors for its development [6, 7].

An earlier study reported that IPF progression is related to epithelial-to-mesenchymal transition (EMT) of alveolar epithelial cells [8]. Through EMT, alveolar epithelial cells capable of regenerating the alveolar architecture transdifferentiate into activated fibroblasts [9]. Therefore, identification of promising EMT-related markers may help us to understand the pathogenesis and progression of IPF.

Circular RNAs (circRNAs) are a class of mainly cytoplasmic non-coding RNAs that have a closed loop structure without 5’ cap or 3’ tail [10]. CircRNAs are enriched in the mammalian tissues, well conserved in sequence, frequently expressed as circRNAs in human, mouse and rat [11–13]. Some circRNAs participate in the regulation of gene expression at the transcriptional or post-transcriptional levels [14], while others appear to regulate gene expression indirectly by regulating RNA polymerase II (Pol II) transcript activity, and circRNAs can compete with the pre-mRNA splicing machinery [15, 16]. Moreover, circRNAs have emerged as novel biomarkers for diagnosis of diseases, including IPF [17]. For instance, downregulation of circHIPK3 reportedly suppresses fibroblast-to-myofibroblast transition in a mouse model of pulmonary fibrosis [10]. However, much remains to be learned about the role of circRNAs in IPF.

In the present study, therefore, bioinformatics analysis using a Gene Expression Omnibus (GEO) dataset was selected to identify differentially expressed circRNAs (DEcircRNAs) in tissues from IPF patients and healthy controls. The results indicate that expression of hsa_circ_0044226 is significantly upregulated in IPF patients. Li et al found that hsa_circ_0044226, also known as hsa_circRNA_102100, was the most strongly upregulated DEcircRNA in IPF tissues, which was consistent with our results [18]. Moreover, the circular RNA interactome (https://circinteractome.nia.nih.gov) indicated that the parental gene for hsa_circ_0044226 is CDC27. Notably, CDC27 protein contributes to EMT in human cancers [19, 20], and EMT also likely contributes to lung fibrosis [21]. We therefore further investigated the role of hsa_circ_0044226 in the development of IPF.

## RESULTS

### Identification of DEcircRNAs in IPF

**Figure 1:**
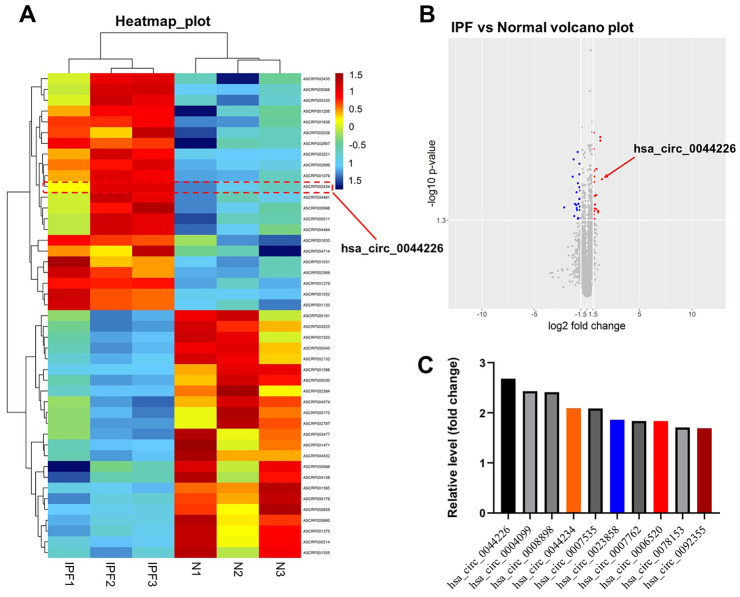
**Identification of DEcircRNAs in IPF.** (**A**) Heat map showing the circRNA expression pattern tissues from IPF patients (IPF group) and healthy controls (N group). (**B**) Volcano plot of DEcircRNAs in GSE102660. The red dots represent the upregulated circRNAs and blue dots represent the upregulated circRNAs. (**C**) The top 10 upregulated DEcircRNAs from IPF tissues and healthy tissues were identified using R language.

**Figure 2:**
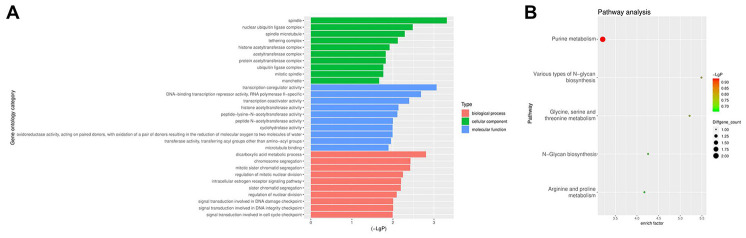
**GO and KEGG analyses of DEcircRNAs.** (**A**) GO enrichment analysis of the parental genes of DEcircRNAs. (**B**) KEGG pathway analysis of the parental genes of DEcircRNAs.

From the GEO database, we analyzed the GSE102660 dataset, which were obtained from tissues from three IPF patients and three healthy controls. Hierarchical clustering analysis revealed circRNA expression patterns that differed between IPF and healthy tissues (Figure 1A). In addition, a total of 45 DEcircRNAs (23 downregulated and 22 upregulated) were identified, and their distribution displayed using a volcano plot (Figure 1B). The top 10 upregulated DEcircRNAs were hsa_circ_0044226, hsa_circ_0004099, hsa_circ_0008898, hsa_circ_0044234, hsa_circ_0007535, hsa_circ_0023858, hsa_circ_0007762, hsa_circ_0006520, hsa_circ_0078153, hsa_circ_0092355 (Figure 1C). Gene Ontology (GO) analysis showed that these DEcircRNAs were mainly enriched in “transcription coregulator activity” and “dicarboxylic and metabolic process” (Figure 2A). Kyoto Encyclopedia of Genes and Genomes (KEGG) analysis revealed that they were mainly enriched in “purine metabolism” (Figure 2B).

### Hsa_circ_0044226 knockdown suppresses TGF-β1-induced migration in RLE-6TN cells

Because hsa_circ_0044226 was the most strongly upregulated DEcircRNA in IPF tissues (Figure 1C) and its parental gene is *cell division cycle 27* (*CDC27*), we selected hsa_circ_0044226 for further study.

**Figure 3:**
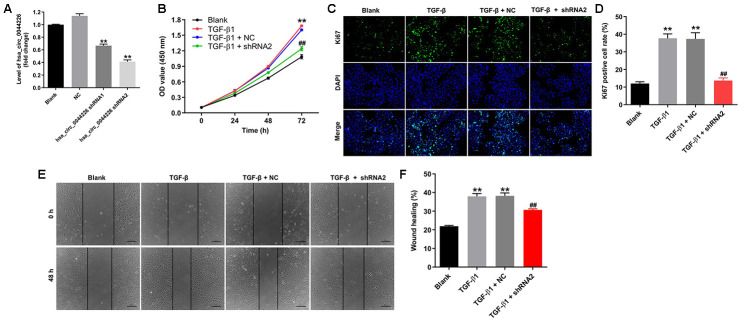
**Hsa_circ_0044226 knockdown suppresses TGF-β1-induced RLE-6TN cell migration.** (**A**) qRT-PCR analysis of hsa_circ_0044226 levels in RLE-6TN cells infected with a lentiviral vector encoding hsa_circ_0044226 shRNA1 or hsa_circ_0044226 shRNA2. (**B**) RLE-6TN cells were pretreated with 10 ng/mL TGF-β1 for 48 h and then transformed with hsa_circ_0044226 shRNA2 for 0, 24, 48 or 72 h. CCK-8 assays were applied to determine the cell viability. (**C**, **D**) RLE-6TN cells were pretreated with 10 ng/mL TGF-β1 for 48 h and then transformed with hsa_circ_0044226 shRNA2 for 72 h. Relative fluorescences were quantified with Ki67 and DAPI staining. (**E**, **F**) Cell migration was detected using wound healing assays. **P < 0.01 vs. Blank group. ^##^P < 0.01 vs. TGF-β1 group.

To investigate the function of hsa_circ_0044226 in RLE-6TN cells, we first knocked it down by transforming the cells using two lentiviral vectors encoding independent shRNAs targeting hsa_circ_0044226. RT-qPCR analysis confirmed that hsa_circ_0044226 levels were suppressed to a greater degree by expression of hsa_circ_0044226 shRNA2 (Figure 3A), which was then utilized in subsequent experiments. In addition, Cell Counting Kit-8 (CCK-8) and Ki67 immunofluorescence assays showed that TGF-β1 promoted the proliferation of RLE-6TN cells, and that effect was significantly inhibited by expression of hsa_circ_0044226 shRNA2 (Figure 3B–3D). TGF-β1 also induced RLE-6TN cell migration, and that effect was largely reversed by hsa_circ_0044226 knockdown (Figure 3E and 3F). Moreover, TGF-β1 remarkedly promoted proliferation and migration of BEAS-2B cells, and these TGF-β1-induced changes in BEAS-2B cells were obviously reversed by hsa_circ_0044226 knockdown (Supplementary Figure 1A and 1B).

### Hsa_circ_0044226 knockdown suppresses TGF-β1-induced EMT in RLE-6TN cells by downregulating CDC27

**Figure 4:**
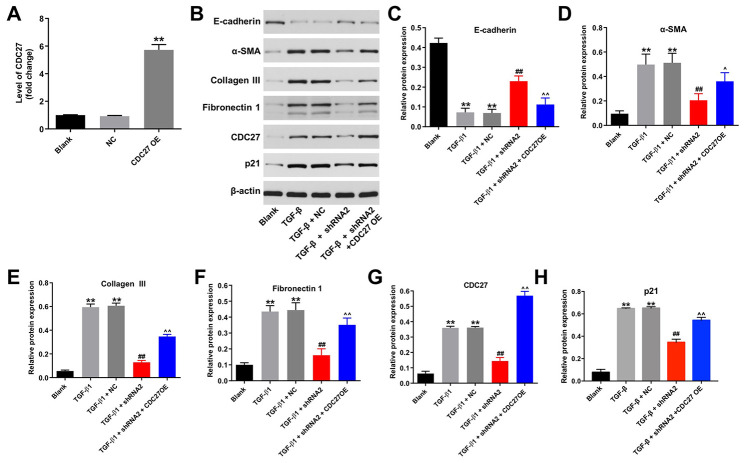
**Hsa_circ_0044226 knockdown suppresses TGF-β1-induced EMT in RLE-6TN cells by downregulating CDC27.** (**A**) qRT-PCR analysis of the levels of CDC27 expression in RLE-6TN cells overexpressing CDC27. (**B**–**H**) RLE-6TN cells were pretreated with 10 ng/mL TGF-β1 for 48 h and then transformed with hsa_circ_0044226 shRNA2 and CDC27 for 72 h. Expression levels of E-cadherin, α-SMA, collagen III, fibronectin 1, CDC27 and p21 were detected with western blotting. Relative expression levels were quantified by normalization to β-actin. **P < 0.01 vs. Blank group. ^##^P < 0.01 vs. TGF-β1 group. ^P < 0.05, ^^P < 0.01 vs. TGF-β1 + shRNA2 group.

To explore the involvement of hsa_circ_0044226 in EMT of RLE-6TN cells and the role of CDC27, RLE-6TN cells were transformed with CDC27 using a lentiviral vector. As indicated in Figure 4A, CDC27 levels were significantly upregulated in RLE-6TN cells after infection with lenti-CDC27. In addition, TGF-β1 markedly reduced levels of E-cadherin expression in RLE-6TN cells while increasing levels of α-SMA, collagen III, fibronectin 1, CDC27 and p21 expression, and these effects were largely reversed by expression of hsa_circ_0044226 shRNA2 (Figure 4B–4H). By contrast, when TG-β1-stimulated RLE-6TN cells were transformed with hsa_circ_0044226 shRNA2 plus CDC27, the effects of hsa_circ_0044226 on expression of E-cadherin, α-SMA, collagen III, fibronectin 1, CDC27 and p21 were reversed by the CDC27 overexpression (Figure 4B–4H).

**Figure 5:**
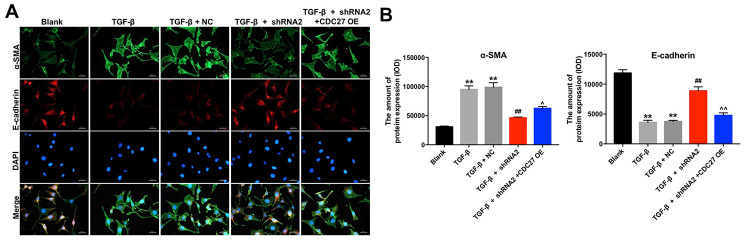
**Hsa_circ_0044226 knockdown suppresses pulmonary fibrosis by inhibiting EMT.** (**A**, **B**) RLE-6TN cells were pretreated with 10 ng/mL TGF-β1 for 48 h and then transformed with hsa_circ_0044226 shRNA2 and CDC27 for 72 h. EMT was assessed based on relative fluorescence of E-cadherin, α-SMA and DAPI staining determined using IHC. **P < 0.01 vs. Blank group. ^##^P < 0.01 vs. TGF-β1 group. ^P < 0.05, ^^P < 0.01 vs. TGF-β1 + shRNA2 group.

IHC analysis confirmed that TGF-β1 significantly reduced expression of E-cadherin and increased the expression of α-SMA in RLE-6TN cells; that the effects of TGF-β1 were largely reversed by hsa_circ_0044226 shRNA2; and that the effects of hsa_circ_0044226 knockdown were in turn reversed by overexpression of CDC27 (Figure 5A and 5B). These results could be confirmed in BEAS-2B cells (Supplementary Figure 1D and 1E). These results suggest that downregulation of hsa_circ_0044226 suppresses TGF-β1-induced EMT in RLE-6TN cells through downregulation of CDC27.

### Hsa_circ_0044226 knockdown alleviates pulmonary fibrosis in a mouse model of IPF

**Figure 6:**
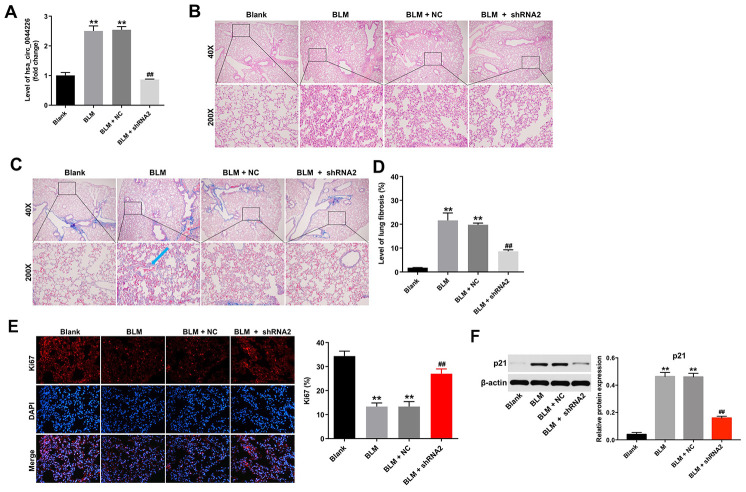
**Hsa_circ_0044226 knockdown alleviates pulmonary fibrosis *in vivo*.** (**A**) RT-qPCR analysis of the levels hsa_circ_0044226 expression in lung tissues from BLM-treated model mice. (**B**, **C**) Collagen deposition and lung fibrosis determined using H&E and Masson’s trichrome staining. Blue arrow, collagen fibers. (**D**) Total lung fibrotic area was measured using Image-Pro Plus. (**E**) IHC staining of ki67 in lung tissues from mice. (**F**) Expression level of p21 was detected with western blotting. Relative expression level was quantified by normalization to β-actin. **P < 0.01 vs. Blank group. ^##^P < 0.01 vs. BLM group.

To further investigate the role of hsa_circ_0044226 in IPF, mice were intratracheally administrated hsa_circ_0044226-shRNA2 immediately after intratracheal administration of bleomycin (BLM). As shown in Figure 6A, levels of hsa_circ_0044226 were significantly increased in the lung tissues of BLM-treated mice, but that effect was greatly inhibited by hsa_circ_0044226-shRNA2 treatment. Staining with H&E and Masson’s trichrome verified that BLM induced a mouse model of lung fibrosis. As shown in Figure 6B-6D, hsa_circ_0044226 knockdown clearly reduced collagen deposition and fibrotic area in BLM-treated mice. In addition, ki67 staining results indicated that BLM significantly inhibited the proliferation of lung epithelial cells in the lung tissues of mice, but that effect was markedly reversed by hsa_circ_0044226-shRNA2 treatment (Figure 6E). Moreover, the expression of p21 was significantly increased in BLM-treated mice, and that effect was remarkedly reversed by hsa_circ_0044226-shRNA2 treatment (Figure 6F).

**Figure 7:**
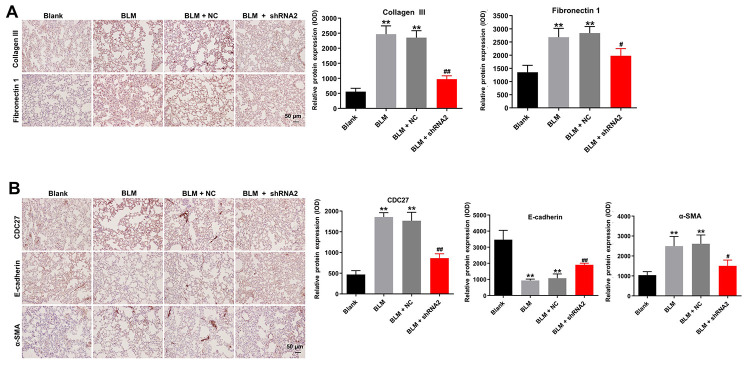
**Hsa_circ_0044226 knockdown alleviates pulmonary fibrosis *in vivo* by suppressing EMT.** (**A**) IHC staining of collagen III, fibronectin in lung tissues from mice. (**B**) IHC staining of α-SMA, E-cadherin, and CDC27 in the lung tissues from mice. **P < 0.01 vs. Blank group. ^#^P < 0.05, ^##^P < 0.01 vs. BLM group.

Furthermore, immunohistochemical analysis demonstrated that hsa_circ_0044226 knockdown abrogated BLM-induced upregulation of fibrosis-related proteins, including collagen III, fibronectin1, α-SMA, and CDC27, in the lung tissues of mice (Figure 7A and 7B). Moreover, knocking down hsa_circ_0044226 also increased the tissue levels of E-cadherin, which were otherwise diminished by BLM administration (Figure 7B). These results suggest that downregulation of hsa_circ_0044226 alleviates pulmonary fibrosis *in vivo* via suppressing the EMT process.

## DISCUSSION

Although circRNAs appear to play important roles in IPF [18], their specific actions remain largely unknown. In this study, we identify that hsa_circ_0044226 is upregulated in BLM-induced pulmonary fibrosis mice model. In addition, downregulation of hsa_circ_0044226 could attenuate pulmonary fibrosis *in vitro* and *in vivo* by inhibiting CDC27.

Regulated in part by TGF-β1 [22, 23], EMT is a process by which epithelial cells acquire some of the traits of mesenchymal cells, including invasiveness and migration [24]. During EMT within the lung, alveolar epithelial cells reportedly acquire various mesenchymal phenotypes, including expression of α-SMA, fibronectin 1 and collagen III, as well as loss of epithelial phenotypes, including expression of E-cadherin [25, 26]. Consistent with those reports, in the present study, TGF-β1-induced EMT in RLE-6TN cells was associated with downregulation of E-cadherin and upregulation of α-SMA, collagen III and fibronectin 1. An earlier study also indicated that suppressing EMT can attenuate pulmonary fibrosis [27]. Similarly, we found that, by inhibiting the EMT process, hsa_circ_0044226 knockdown inhibited TGF-β1-induced fibrosis in RLE-6TN cells as well as BLM-induced pulmonary fibrosis in model mice. It thus appears that knocking down hsa_circ_0044226 attenuates pulmonary fibrosis *in vitro* and *in vivo* by inhibiting EMT.

CDC27 is a core subunit of anaphase-promoting complex/cyclosome (APC/C) [28]. APC/C is a type of E3 ligase complex that regulates chromosome segregation and mitotic exit [29]. APC/C reportedly participates in the TGF-β signaling pathway, and TGF-β activates CDC27 [30]. In addition, overexpression of CDC27 induces invasion and migration by gastric cancer cells, which is associated with downregulation of the EMT-related biomarker E-cadherin and upregulation of vimentin [19]. In the present study, TGF-β1 significantly increased levels of CDC27 in RLE-6TN cells, while hsa_circ_0044226 knockdown substantially reduced CDC27 expression in TGF-β1-treated RLE-6TN cells and in a mouse pulmonary fibrosis model, and those effects were largely reversed by exogenous expression of CDC27. This suggests overexpression of CDC27 abolishes the antifibrotic effect of hsa_circ_0044226 knockdown on TGF-β1-treated RLE-6TN cells through activation of EMT. Zhang et al found that circHIPK2 could promote fibroblast proliferation via a ceRNA-mediated mechanism, in which circHIPK3 functions as an endogenous miR-338-3p sponge, leading to upregulated SOX4 expression [10]. Unlike the classical ceRNA mechanism, fang et al found that circHECTD1 could promote lung fibroblast-to-myofibroblast transition regulate the expression of its host gene, HECTD1 [31]. In this study, we found that hsa_circ_0044226 could regulate the expression of its host gene, CDC27, through affecting gene transcription, although further studies are needed to verify this hypothesis. Collectively then, these findings indicate that downregulation of hsa_circ_0044226 attenuates pulmonary fibrosis *in vitro* and *in vivo* by inhibiting CDC27, which in turn suppresses EMT. Hsa_circ_0044226 is thus a potentially useful therapeutic target for the treatment of IPF. However, the disadvantage is that only 3 samples of IPF tissue and normal tissues was analyzed in this study. In the future, we need to collect the substantial amount of IPF samples for exploring therapeutic strategies for IPF.

## MATERIALS AND METHODS

### CircRNA data analysis and bioinformatics

The GSE102660 dataset, which contains the circRNA expression data for tissues from IPF patients and healthy controls, was downloaded from the GEO database (https://www.ncbi.nlm.nih.gov/geo/). The R language was then used to screen the DEcircRNAs. The criteria for selection of DEcircRNAs were a -log10 P-value > 1.3 and a |fold change| >1.5. The functions and signaling pathway of the genes encoding the DEcircRNAs were analyzed using GO (http://www.geneontology.org/) and KEGG (http://www.genome.jp/kegg/) enrichment analysis, respectively.

### Cell culture

Alveolar type II epithelial (RLE-6TN) cells and human bronchial epithelial cells (BEAS-2B) were purchased from the American Type Culture Collection (ATCC, Rockville, Maryland, USA) and were maintained in Dulbecco’s modified Eagle’s medium (DMEM) supplemented with 10% fetal bovine serum, 100 U/ml penicillin, 100 μg/ml streptomycin under 5% CO_2_ atmosphere with 95% humidity at 37°C. Some cells were cultured in medium containing TGF-β1 (final concentration, 10 ng/mL; Sigma Aldrich, St. Louis, MO, USA) for 48 h before further analysis.

### Lentivirus production and stable cell lines construction

Two pairs of cDNA oligonucleotides encoding short hairpin RNAs inhibiting hsa_circ_0044226 expression were inserted into the pLVX-shRNA plasmid (GenePharma, Shanghai, China), yielding hsa_circ_0044226-shRNA1 and hsa_circ_0044226-shRNA2. In addition, a lentiviral construct for CDC27 overexpression (lenti-CDC27) was obtained from GenePharma. 293T cells were transformed with the hsa_circ_0044226-shRNA1, hsa_circ_0044226-shRNA2 or lenti-CDC27 plasmid and incubated for 72 h. The supernatants from these cells were then collected and used to infect RLE-6TN cells for 24 h, after which they were incubated for 48 h with puromycin (2.5 μg/mL, Thermo Fisher Scientific, Waltham, MA, USA) to select stable hsa_circ_0044226 knockdown or overexpressing cells. Stable overexpression or knockdown of hsa_circ_0044226 was confirmed using reverse transcription quantitative polymerase chain reaction (qRT-PCR).

The sequences of hsa_circ_0044226-shRNA1 were 5’-GATCGCCATCTACCTTCTCACCACT TTCAAGAGAAGTGGTGAGAAGGTAGATGGCTTTTTTGGTACC-3’ (forward) and 5’AATTGGTACCAAAAAAGCCATCTACCTTCTCACCACTTCTCTTGAAAGTG GTGAGAAGGTAGATGGC-3’ (reverse). The sequences of hsa_circ_0044226-shRNA2 were 5’-GAT CGCAACGGGAACATGATATTGCTTCAAGAGAGCAATATCATGTTCCCGTTGCTTTTTTGGTACC-3’ (forward) and 5’-AATTGGTACCAAAAAA GCAACGGGAACATGATATTGCTCTCTTGAAGCAATATCATGTTCCCGTTGC-3’ (reverse).

### RT-qPCR

TRIpure Total RNA Extraction Reagent (ELK Biotechnology, Wuhan, China) was used to extract total RNAs from RLE-6TN cells, after which the RNA samples were reverse transcribed to cDNAs using an EntiLink™ 1^st^ Strand cDNA Synthesis Kit (ELK Biotechnology) according to the manufacturer’s protocol. qPCR analysis was then performed using EnTurbo™ SYBR Green PCR SuperMix (ELK Biotechnology) on an Agilent Technologies Stratagene Mx3000p Real-Time System (Agilent Technologies, Inc., San Francisco, USA). The primer sequences were: for actin, 5’- CGTTGACATCCGTAAAGACCTC-3’ (forward) and 5’-TAGGAGCCAGGGCAGTAATCT-3’ (reverse); for CDC27, 5’- TGCTATATGGCAAGCACTAAACC-3’ (forward) and 5’- CATTTTGCAAGCAGGTATTTACAC-3’ (reverse); and for Hsa_circ_0044226, 5’- ATGCATGTACAACACCTCAGGTAT-3’ (forward) and 5’-CTTCTGAAATGATGGAAGAGTCC-3’ (reverse). Actin served as a control for the quantitation of RNA using the comparative threshold cycle (2^−ΔΔCT^) method.

### Cell proliferation assay

RLE-6TN cells were plated onto 96-well plates (10^4^ cells/well) and incubated overnight at 37°C. The cells were then pretreated with 10 ng/mL TGF-β1 for 48 h and infected with hsa_circ_0044226-shRNA2 for 72 h. For cell counts, 10 μL of the CCK-8 reagent (Beyotime, Shanghai, China) was added into each well, and the cells were incubated for an additional 2 h incubation at 37°C. The absorbance at 450 nm was then measured using a microplate reader (Bio-Rad Laboratories, Inc., Hercules, CA, USA).

### Immunofluorescent staining

RLE-6TN cells were fixed in PBS containing 4% paraformaldehyde and then blocked for 2 h in 10% goat serum (Abcam Cambridge, MA, USA). Thereafter, the cells were incubated first with primary antibody against Ki67 (1: 100, Abcam), α-SMA (1: 100, Abcam), or E-cadherin (1: 100, Abcam) overnight at 4°C, and then with the horseradish peroxidase-conjugated secondary antibodies at room temperature for 1 h. After staining the nuclei with DAPI for 5 min, the cells were observed under a laser scanning confocal microscope (Olympus CX23 Tokyo, Japan). Ki67-, α-SMA- and E-cadherin-positive cells were analyzed from at least three fields using Image pro-plus.

### Wound healing assay

RLE-6TN cells were plated onto 12-well culture plates (5×10^5^ cells per well) and incubated overnight at 37 °C, after which wounds on the endothelial monolayers were made using a 200-μL pipette tip. The cells were then infected with hsa_circ_0044226-shRNA2 for 24 h. The wounds were monitored by photographing them at 0 h and 24 h using a fluorescence microscope (Olympus). ImageJ software was used to measure the area of cell migration.

### Western blot assay

Total proteins were quantified using the BCA method (Beyotime Institute of Biotechnology), after which aliquots were separated on 10% SDS-PAGE and then transferred onto polyvinylidene difluoride membranes (Thermo Fisher Scientific). After blocking with 5% skimmed milk in Tris-buffered saline plus 0.1% Tween-20 (TBST) for 1 h, the membranes were incubated first with primary antibody against E-cadherin (1:1000, Abcam), α-SMA (1:1000, Abcam), collagen III (1:1000, Abcam), fibronectin 1 (1:1000, Abcam), CDC27 (1:1000, Abcam), and β-actin (1:1000, Abcam) at 4°C overnight, and then with horseradish peroxidase-conjugated secondary antibodies (Abcam, 1: 5000) at room temperature for 1 h. Finally, an electrochemiluminescence detection system (Thermo Fisher Scientific) was used to detect the immunoreactive signals. β-actin served as an internal control.

### Animal studies

A total of 20 male C57BL/6 mice (9-12 weeks old) were purchased from Beijing Vital River Experimental Animal Technology Co., Ltd (Beijing, China) and kept under standard animal room conditions following the guidelines of the Institutional Animal Care and Use Committee. All animal experiments were performed according to the rules approved by the Institutional Ethical Committee of the experimental animal center of Peking University First Hospital. The mice were randomly divided into four groups: Blank, BLM, BLM + NC, and BLM + hsa_circ_0044226-shRNA2. To induce fibrotic changes, mice under anesthesia were given a single intratracheal injection of sterile saline containing BLM (50 μL, 1 U/kg). Hsa_circ_0044226-shRNA2 was administered after BLM treatment via intratracheal injection. Four weeks later, the mice were sacrificed, and the lung tissues were collected, fixed in 4% paraformaldehyde, and embedded in paraffin.

### Histology and immunohistochemistry (IHC)

For histological analysis to assess lung fibrosis, consecutive 5-μm sections were cut from the lung tissues, and sections were stained with hematoxylin-eosin (H&E) or Masson’s trichrome, and observed using the fluorescence microscope (Olympus). For immunohistochemical analysis of Ki67, E-cadherin, α-SMA, collagen III, fibronectin 1, and CDC27 expression, the sections were stained using IHC as described previously [32]. Briefly, the sections were incubated first with primary antibodies overnight at 4°C, and then with biotinylated goat anti-rabbit IgG for 30 min at room temperature. An IHC detection system (EnVision kit; Dako Japan) was used for visualization. The cells with positive staining of Ki67, E-cadherin, α-SMA, collagen III, fibronectin 1, and CDC27 were counted from three randomly selected areas using Image pro-plus.

### Statistical analysis

All experiments were performed in triplicate. Data are presented as the mean ± SD. All statistical analyses were performed using GraphPad Prism software (version 7.0, La Jolla, CA, USA). One-way analysis of variance (ANOVA) and Tukey’s tests were used to make multiple group comparisons. Values of P < 0.05 were considered significant.

## Supplementary Material

Supplementary Figure 1

## References

[1] Li LC, Kan LD. Traditional chinese medicine for pulmonary fibrosis therapy: progress and future prospects. J Ethnopharmacol. 2017; 198:45–63. 10.1016/j.jep.2016.12.042 28038955 PMC7127743

[2] Cho SJ, Hong KS, Jeong JH, Lee M, Choi AM, Stout-Delgado HW, Moon JS. DROSHA-dependent AIM2 inflammasome activation contributes to lung inflammation during idiopathic pulmonary fibrosis. Cells. 2019; 8:938. 10.3390/cells8080938 31434287 PMC6721825

[3] Yao MY, Zhang WH, Ma WT, Liu QH, Xing LH, Zhao GF. microRNA-328 in exosomes derived from M2 macrophages exerts a promotive effect on the progression of pulmonary fibrosis via FAM13A in a rat model. Exp Mol Med. 2019; 51:1–16. 10.1038/s12276-019-0255-x 31164635 PMC6547742

[4] Sgalla G, Iovene B, Calvello M, Ori M, Varone F, Richeldi L. Idiopathic pulmonary fibrosis: pathogenesis and management. Respir Res. 2018; 19:32. 10.1186/s12931-018-0730-2 29471816 PMC5824456

[5] Fernandez IE, Eickelberg O. New cellular and molecular mechanisms of lung injury and fibrosis in idiopathic pulmonary fibrosis. Lancet. 2012; 380:680–88. 10.1016/S0140-6736(12)61144-1 22901889

[6] Baumgartner KB, Samet JM, Stidley CA, Colby TV, Waldron JA. Cigarette smoking: a risk factor for idiopathic pulmonary fibrosis. Am J Respir Crit Care Med. 1997; 155:242–48. 10.1164/ajrccm.155.1.9001319 9001319

[7] Zaman T, Lee JS. Risk factors for the development of idiopathic pulmonary fibrosis: a review. Curr Pulmonol Rep. 2018; 7:118–25. 10.1007/s13665-018-0210-7 31588408 PMC6777743

[8] Tian R, Zhu Y, Yao J, Meng X, Wang J, Xie H, Wang R. NLRP3 participates in the regulation of EMT in bleomycin-induced pulmonary fibrosis. Exp Cell Res. 2017; 357:328–34. 10.1016/j.yexcr.2017.05.028 28591554

[9] Harada T, Nabeshima K, Hamasaki M, Uesugi N, Watanabe K, Iwasaki H. Epithelial-mesenchymal transition in human lungs with usual interstitial pneumonia: quantitative immunohistochemistry. Pathol Int. 2010; 60:14–21. 10.1111/j.1440-1827.2009.02469.x 20055947

[10] Zhang JX, Lu J, Xie H, Wang DP, Ni HE, Zhu Y, Ren LH, Meng XX, Wang RL. circHIPK3 regulates lung fibroblast-to-myofibroblast transition by functioning as a competing endogenous RNA. Cell Death Dis. 2019; 10:182. 10.1038/s41419-019-1430-7 30796204 PMC6385182

[11] Rybak-Wolf A, Stottmeister C, Glažar P, Jens M, Pino N, Giusti S, Hanan M, Behm M, Bartok O, Ashwal-Fluss R, Herzog M, Schreyer L, Papavasileiou P, et al. Circular RNAs in the mammalian brain are highly abundant, conserved, and dynamically expressed. Mol Cell. 2015; 58:870–85. 10.1016/j.molcel.2015.03.027 25921068

[12] Werfel S, Nothjunge S, Schwarzmayr T, Strom TM, Meitinger T, Engelhardt S. Characterization of circular RNAs in human, mouse and rat hearts. J Mol Cell Cardiol. 2016; 98:103–07. 10.1016/j.yjmcc.2016.07.007 27476877

[13] Pamudurti NR, Bartok O, Jens M, Ashwal-Fluss R, Stottmeister C, Ruhe L, Hanan M, Wyler E, Perez-Hernandez D, Ramberger E, Shenzis S, Samson M, Dittmar G, et al. Translation of CircRNAs. Mol Cell. 2017; 66:9–21.e7. 10.1016/j.molcel.2017.02.021 28344080 PMC5387669

[14] Kristensen LS, Hansen TB, Venø MT, Kjems J. Circular RNAs in cancer: opportunities and challenges in the field. Oncogene. 2018; 37:555–65. 10.1038/onc.2017.361 28991235 PMC5799710

[15] Shan C, Zhang Y, Hao X, Gao J, Chen X, Wang K. Biogenesis, functions and clinical significance of circRNAs in gastric cancer. Mol Cancer. 2019; 18:136. 10.1186/s12943-019-1069-0 31519189 PMC6743094

[16] Ashwal-Fluss R, Meyer M, Pamudurti NR, Ivanov A, Bartok O, Hanan M, Evantal N, Memczak S, Rajewsky N, Kadener S. circRNA biogenesis competes with pre-mRNA splicing. Mol Cell. 2014; 56:55–66. 10.1016/j.molcel.2014.08.019 25242144

[17] Yang L, Liu X, Zhang N, Chen L, Xu J, Tang W. Investigation of circular RNAs and related genes in pulmonary fibrosis based on bioinformatics analysis. J Cell Biochem. 2019; 120:11022–32. 10.1002/jcb.28380 30767300 PMC6593700

[18] Li R, Wang Y, Song X, Sun W, Zhang J, Liu Y, Li H, Meng C, Zhang J, Zheng Q, Lv C. Potential regulatory role of circular RNA in idiopathic pulmonary fibrosis. Int J Mol Med. 2018; 42:3256–68. 10.3892/ijmm.2018.3892 30272257 PMC6202105

[19] Xin Y, Ning S, Zhang L, Cui M. CDC27 Facilitates Gastric Cancer Cell Proliferation, Invasion and Metastasis via Twist-Induced Epithelial-Mesenchymal Transition. Cell Physiol Biochem. 2018; 50:501–511. 10.1159/000494164 30308498

[20] Qiu L, Tan X, Lin J, Liu RY, Chen S, Geng R, Wu J, Huang W. CDC27 induces metastasis and invasion in colorectal cancer via the promotion of epithelial-to-mesenchymal transition. J Cancer. 2017; 8:2626–35. 10.7150/jca.19381 28900500 PMC5595092

[21] Kage H, Borok Z. EMT and interstitial lung disease: a mysterious relationship. Curr Opin Pulm Med. 2012; 18:517–23. 10.1097/MCP.0b013e3283566721 22854509 PMC3914631

[22] Samarakoon R, Overstreet JM, Higgins PJ. TGF-β signaling in tissue fibrosis: redox controls, target genes and therapeutic opportunities. Cell Signal. 2013; 25:264–68. 10.1016/j.cellsig.2012.10.003 23063463 PMC3508263

[23] Li J, Feng M, Sun R, Li Z, Hu L, Peng G, Xu X, Wang W, Cui F, Yue W, He J, Liu J. Andrographolide ameliorates bleomycin-induced pulmonary fibrosis by suppressing cell proliferation and myofibroblast differentiation of fibroblasts via the TGF-β1-mediated Smad-dependent and -independent pathways. Toxicol Lett. 2020; 321:103–113. 10.1016/j.toxlet.2019.11.003 31706003

[24] Acloque H, Adams MS, Fishwick K, Bronner-Fraser M, Nieto MA. Epithelial-mesenchymal transitions: the importance of changing cell state in development and disease. J Clin Invest. 2009; 119:1438–49. 10.1172/JCI38019 19487820 PMC2689100

[25] Bang IJ, Kim HR, Jeon Y, Jeong MH, Park YJ, Kwak JH, Chung KH. Β-peltoboykinolic acid from Astilbe rubra attenuates TGF-β1-induced epithelial-to-mesenchymal transitions in lung alveolar epithelial cells. Molecules. 2019; 24:2573. 10.3390/molecules24142573 31311194 PMC6680586

[26] Salton F, Volpe MC, Confalonieri M. Epithelial-Mesenchymal transition in the pathogenesis of idiopathic pulmonary fibrosis. Medicina (Kaunas). 2019; 55:83. 10.3390/medicina55040083 30925805 PMC6524028

[27] Song L, Sen S, Sun Y, Zhou J, Mo L, He Y. Ketamine Inhalation Ameliorates Ovalbumin-Induced Murine Asthma by Suppressing the Epithelial-Mesenchymal Transition. Med Sci Monit. 2016; 22:2471–83. 10.12659/msm.899955 27418244 PMC4958373

[28] Chang L, Zhang Z, Yang J, McLaughlin SH, Barford D. Atomic structure of the APC/C and its mechanism of protein ubiquitination. Nature. 2015; 522:450–54. 10.1038/nature14471 26083744 PMC4608048

[29] Schreiber A, Stengel F, Zhang Z, Enchev RI, Kong EH, Morris EP, Robinson CV, da Fonseca PC, Barford D. Structural basis for the subunit assembly of the anaphase-promoting complex. Nature. 2011; 470:227–32. 10.1038/nature09756 21307936

[30] Zhang L, Fujita T, Wu G, Xiao X, Wan Y. Phosphorylation of the anaphase-promoting complex/Cdc27 is involved in TGF-beta signaling. J Biol Chem. 2011; 286:10041–50. 10.1074/jbc.M110.205518 21209074 PMC3060455

[31] Fang S, Guo H, Cheng Y, Zhou Z, Zhang W, Han B, Luo W, Wang J, Xie W, Chao J. circHECTD1 promotes the silica-induced pulmonary endothelial-mesenchymal transition via HECTD1. Cell Death Dis. 2018; 9:396. 10.1038/s41419-018-0432-1 29540674 PMC5852113

[32] Henri O, Pouehe C, Houssari M, Galas L, Nicol L, Edwards-Lévy F, Henry JP, Dumesnil A, Boukhalfa I, Banquet S, Schapman D, Thuillez C, Richard V, et al. Selective stimulation of cardiac lymphangiogenesis reduces myocardial edema and fibrosis leading to improved cardiac function following myocardial infarction. Circulation. 2016; 133:1484–97. 10.1161/CIRCULATIONAHA.115.020143 26933083

