# Supplementary material for: Hsa_circ_0044226 knockdown attenuates progression of pulmonary fibrosis by inhibiting CDC27
Source: Aging (Albany NY). 2020 Jul 24;12(14):14808–18. doi: 10.18632/aging.103543 (PMC7425454; doi:10.18632/aging.103543)
Supplement: Supplementary Figure 1 [file aging-12-103543-s001..pdf]

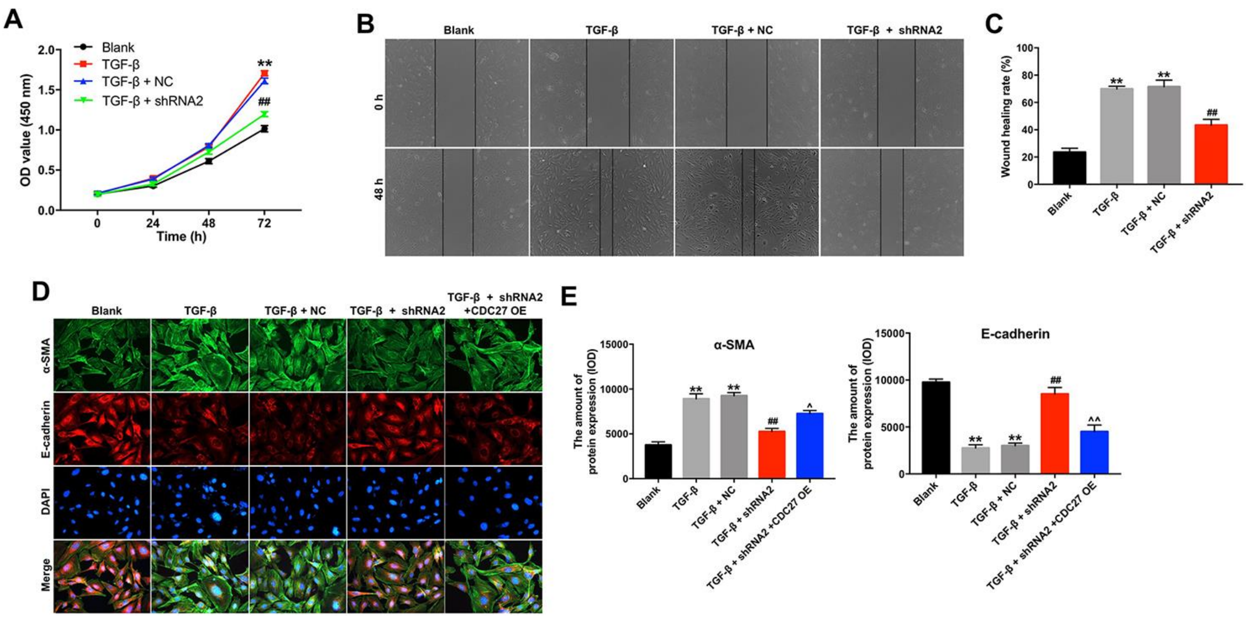

Supplementary Figure 1. Hsa_circ_0044226 knockdown suppresses TGF-β1-induced EMT in BEAS-2B cells by downregulating CDC27. (**A**) BEAS-2B cells were pretreated with 10 ng/mL TGF-β1 for 48 h and then transformed with hsa_circ_0044226 shRNA2 for 0, 24, 48 or 72 h. CCK-8 assays were applied to determine the cell viability. (**B**, **C**) Cell migration was detected using wound healing assays. (**D**, **E**) EMT was assessed based on relative fluorescence of E-cadherin, α-SMA and DAPI staining determined using IHC. $^{**}P < 0.01$ vs. Blank group. $^{\#\#}P < 0.01$ vs. TGF-β1 group. $^{\wedge}P < 0.05$, $^{\wedge\wedge}P < 0.01$ vs. TGF-β1 + shRNA2 group.
